# Social network enhanced behavioral interventions for diabetes and obesity: A 3 arm randomized trial with 2 years follow-up in Jordan

**DOI:** 10.1371/journal.pgph.0001514

**Published:** 2024-03-20

**Authors:** Eric L. Ding, Andrea B. Feigl, Kathleen T. Watson, Tin Lok James Ng, Leila Makerechi, Nancy Bui, Amal Ireifij, Rami Farraj, Daniel E. Zoughbie

**Affiliations:** 1 New England Complex Systems Institute, Public Health, Cambridge, Massachusetts, United States of America; 2 Microclinic International, Social Network Research Group, San Francisco, California, United States of America; 3 Harvard T.H. Chan School of Public Health (Previous Affiliation of ABF, ELD), Boston, Massachusetts, United States of America; 4 Health Finance Institute, Washington, D.C. United States of America; 5 Behavioral Sciences, Stanford University, Stanford, California, United States of America; 6 School of Computer Science and Statistics, Trinity College Dublin, Dublin, Ireland; 7 Jordanian Royal Health Awareness Society, Amman, Kingdom of Jordan; 8 Institute of International Studies, University of California, Berkeley, Berkeley, California, United States of America; University of the Witwatersrand, SOUTH AFRICA

## Abstract

While obesity and diabetes are rising pandemics, few low-cost and effective prevention and management strategies exist, especially in the Middle East. Nearly 20% of adults in Jordan suffer from diabetes, and over 75% are overweight or obese. Social network-based programs have shown promise as a viable public health intervention strategy to address these growing crises. We evaluated the effectiveness of the Microclinic Program (MCP) via a 6-month multi-community randomized trial in Jordan, with follow-up at 2 years. The MCP leverages existing social relationships to propagate positive health behaviors and information. We recruited participants from 3 community health centers in Amman, Jordan. Participants were eligible for the study if they had diabetes, pre-diabetes, or possessed ≥1 metabolic risk factor along with a family history of diabetes. We randomized participants into three trial arms: (A Group) received the Full MCP with curriculum-activated social network interactions; (B Group) received Basic MCP educational sessions with organic social network interactions; or (C Group-Control) received standard care coupled with active monitoring and parallel screenings. Groups of individuals were randomized as units in a 3:1:1 ratio, with resulting group sizes of n = 540, 186, and 188 in arms A, B, and C, respectively. We assessed the overall changes in body weight, fasting glucose, hemoglobin A1c (HbA1c) and mean arterial blood pressure between study arms in multiple evaluations across 2 years (including at 6-months and 2-years follow-up). We investigated the effectiveness of Full and Basic MCP social network interventions using multilevel models for longitudinal data with hierarchical nesting of individuals within MCP classrooms, within community centers, and within temporal cohorts. We observed significant overall 2-year differences between all 3 groups for changes in body weight (P = 0.0003), fasting blood glucose (P = 0.0015), and HbA1c (P = 0.0004), but not in mean arterial blood pressure (P = 0.45). However, significant changes in mean arterial pressure were observed for Full MCP versus controls (P = 0.002). Weight loss in the Full MCP exceeded (-0.97 kg (P<0.001)) the Basic MCP during the intervention. Furthermore, both Full and Basic MCP yielded greater weight loss compared to the control group at 2 years. The Full MCP also sustained a superior fasting glucose change over 2 years (overall P<0.0001) versus the control group. For HbA1c, the Full MCP similarly led to greater 6-month reduction in HbA1c versus the control group (P<0.001), with attenuation at 2 years. For mean arterial blood pressure, the Full MCP yielded a greater drop in blood pressure versus control at 6 months; with attenuation at 2 years. These results suggest that activated social networks of classroom interactions can be harnessed to improve health behaviors related to obesity and diabetes. Future studies should investigate how public health policies and initiatives can further leverage social network programs for greater community propagation.

**Trial registration.** ClinicalTrials.gov Identifier: NCT01818674.

## Introduction

Global annual diabetes costs are estimated at $825 billion [1]. The Middle East contributes a large percentage of the global non-communicable disease (NCD) burden [2, 3], with diabetes burden increasing in the Middle East region by 216% between 1990 to 2015 alone [4]. In Jordan, NCDs were responsible for more than 78% of all deaths in 2018, and that percentage continues to rise [5–7]. High body-mass index (BMI), fasting plasma glucose (FPG), dietary risks, blood pressure (BP), smoking, and LDL were the top causes of disability-adjusted life years (DALYs). Notably, the proportion of DALYs attributed to high BMI more than doubled from 3.7% to 7.5% between 1990 and 2013 in the Middle East [8].

Despite the substantial disease and economic burden [9] of NCD, national NCD prevention strategies in the Middle East, and Jordan specifically, are sparse [2, 10]. In 2008, Ajlouni et al. published a cross-sectional sample that randomly sampled patients, finding that 54% of individuals who have diabetes in Jordan received insufficient care [5]. Chronic disease risk factor self-management in Jordan is poor, and wanes with increasing time post-diagnosis [5, 11, 12].

Public health studies have established the role of social networks in spreading attitudes and behaviors associated with both infectious diseases such as COVID-19 [13], as well as chronic diseases and their prevention [14, 15]. However, most of these studies have used passive observational designs, non-clinical endpoints, do not allow differentiation between social network modalities, or do not include an intervention [16, 17].

One intervention, the Microclinic Program (MCP) has been designed to address these issues [18–29]. After completing a successful cohort analysis of a 4-month program and followed for 2 years demonstrating sustained weight-loss and improvements in blood glucose and cholesterol among diabetic and at-risk Jordanians [30], and after conducting a 2-arm trial in Appalachia-Kentucky [19, 20], the MCP program was adopted by the United Nation’s Relief Works Agency as its core diabetes management program for refugees in Jordan, West Bank, Gaza, and Lebanon [27].

In efforts to further demonstrate the effects of an enhanced social-network MCP intervention, we conducted a three-arm RCT to further test the impact of a more advanced 6-month MCP intervention with 2-year follow-up on diabetes and obesity management on metabolic outcomes in Amman, Jordan. Our hypothesis was that individuals who received the full MCP social network behavioral health program would experience greater health improvements relative to a basic MCP behavioral health educational program or a standard care program coupled with active monitoring and parallel screenings.

## Methods

### Study procedures

#### Design and setting

We conducted this trial (US Clinical Trials Registry Number: NCT01818674) in collaboration with Queen Rania’s Royal Health Awareness Society and the Jordanian Ministry of Health (MoH). The three-arm RCT was rolled out in four waves at three participating centers located in three neighborhoods in Amman, Jordan. Participants were recruited through MoH centers using a combination of outreach campaigns and center patient recruitment between October 2011 and May 2013. Individuals who indicated interest in participating were invited to attend a study information session and an eligibility criteria confirmation appointment at one of the centers.

Men and women 18 years or older were eligible to participate in the study if they had been previously diagnosed with diabetes, were diagnosed with diabetes or pre-diabetes during recruitment, or were at risk of diabetes, meaning they had at least one risk factor for diabetes. Diabetes and pre-diabetes were confirmed with a fasting plasma glucose (FPG) test at recruitment, using criteria of 100–125 mg/dL for pre-diabetes, and 126 mg/dL or higher for diabetes. Risk of diabetes was defined as having a history of diabetes in close family members AND being overweight/obese, or as having a family history of diabetes AND having either high BP or high serum cholesterol. Pregnant and/or severely ill participants were not eligible.

#### Randomization procedures

Randomization was stratified by study center (three centers), and study cohort waves (up to four), resulting in a total of nine study center-cohorts. “Units” of individuals who attended screenings alone and “units” of multiple individuals comprised of family and friends who attended screenings together, and were randomized to one of the three arms together, regardless of whether they came to screenings as singles or groups. All randomized units met inclusion criteria.

Individuals who were eligible went through randomization process and were designated as “nodes,” regardless of whether they were randomized as solo individuals, or as a special unit together if they were family and friends. We randomized the units into classrooms that received one of the 3 interventions; these classrooms were stable groups who participated together weekly throughout the intervention period in their respective assigned randomized arm. In the Full MCP intervention arm (Arm A), units that were initially solo individuals were further encouraged to form a friendship circle with another unit in the same classroom; such friendship circles were called “Microclinics”. In the Basic MCP arm (Arm B) and in the control arm (Arm C), microclinic groups consisted only of individuals who initially came as friends and family before randomization and did not include structural formation of any new friendship groups post-randomization. Microclinics consisted of 2 to 8 individuals nested within MCP classrooms, which consisted of up to 26 individuals. These classrooms, in turn, were nested within 3 community centers, and all of these were further nested within 4 temporal cohorts.

Furthermore, in the Full MCP program, we included the adjunct support of individuals who did not enter the initial randomization process with a friend/family unit, but instead were later invited post-randomization to participate in the educational programs of the classroom in a supportive role alongside the nodes as friends/families. These supporting individuals attended and participated in Arm A program activities and were dubbed as "secondaries" and were part of social circles designated as “Expanded Microclinics”. To preserve randomization in the trial, only initially recruited nodes were analyzed as part of our randomized trial analysis (i.e. data from secondaries were therefore excluded).

#### Study intervention

A “Microclinic” is social infrastructure, not a physical structure. They are groups of friends and family who transform private spaces into spaces of health. Together, Microclinic participants influence one another to eat healthy, exercise, monitor, and adhere to medication. Accordingly, MCP’s educational lessons are summarized by “4 M’s”: Meals, Movement, Monitoring, Medication.

The primary MCP interventions were delivered over a timeframe of 6 months (28 weeks) and involved 14 program sessions (Arms A and B), or 14 concurrent check-in appointments (Arm C), all at parallel time intervals between arms, with the first intervention cohort starting in January 2012. Follow-up post-intervention data was collected at approximately 2 years, comprised of data collected between 21–28 months (median 24 months) after baseline.

Intervention Arm A received the Full MCP educational program. In addition, they worked within small Microclinic groups to complete behavior change assignments inside and outside classrooms. Before each class began, Arm A was taught a social network-based theory of change as follows: 1) I influence and am influenced by the behaviors of those around me, 2) this can be good or bad, 3) I can improve my own behaviors as well as those in my community.

Intervention Arm B consisted of the Basic MCP intervention in which participants received the same educational instructions as Arm A. Given many people in the program knew each other socially or were related to one another from the wider neighborhood, organic classroom social interactions were inevitable. However, Basic MCP classroom participants were not assigned to structured small group Microclinic activities. Classroom discussions were also not initiated by the facilitators, though they were self-initiated by participants.

The fundamental distinction between intervention Arms A and B consisted of the degree to which group social interaction was embedded in the design of activities and the way this socialization occurred. While both Arms A and B covered the same diabetes educational content, notably the four M’s, Arm A was structured to actively promote group-based socialization among friends, family, and classrooms, whereas Arm B allowed this socialization to occur more organically. See S1 and S2 Tables for further programming differences between arms. Arm C, the control group, did not receive any classes; however, participants attended parallel appointments on any weekday, within the same matching week as concurrently run Arm A and B classroom programs, to individually collect their biometric data and lab measurements without any other interactions or programming.

#### Sample size

**Fig 1:**
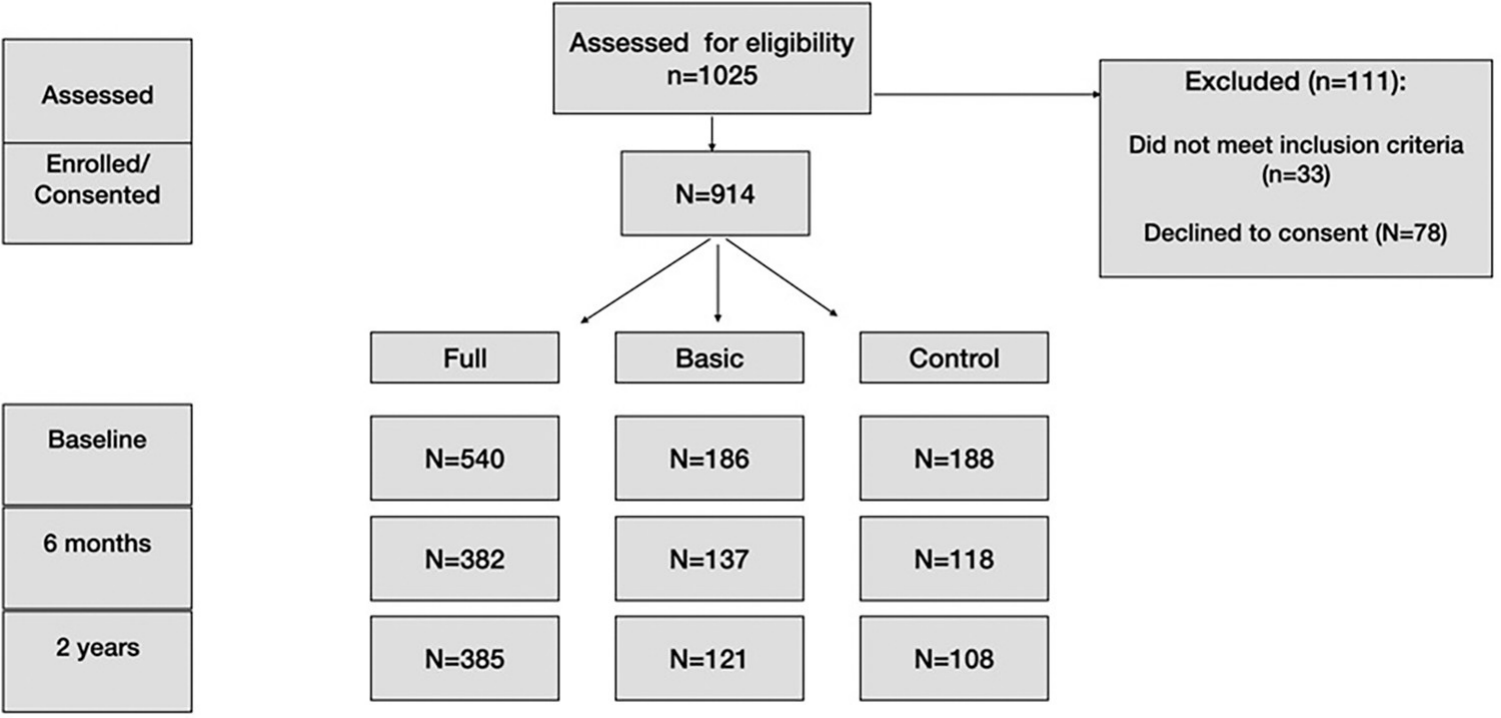
Participant flow diagram.

**Fig 2:**
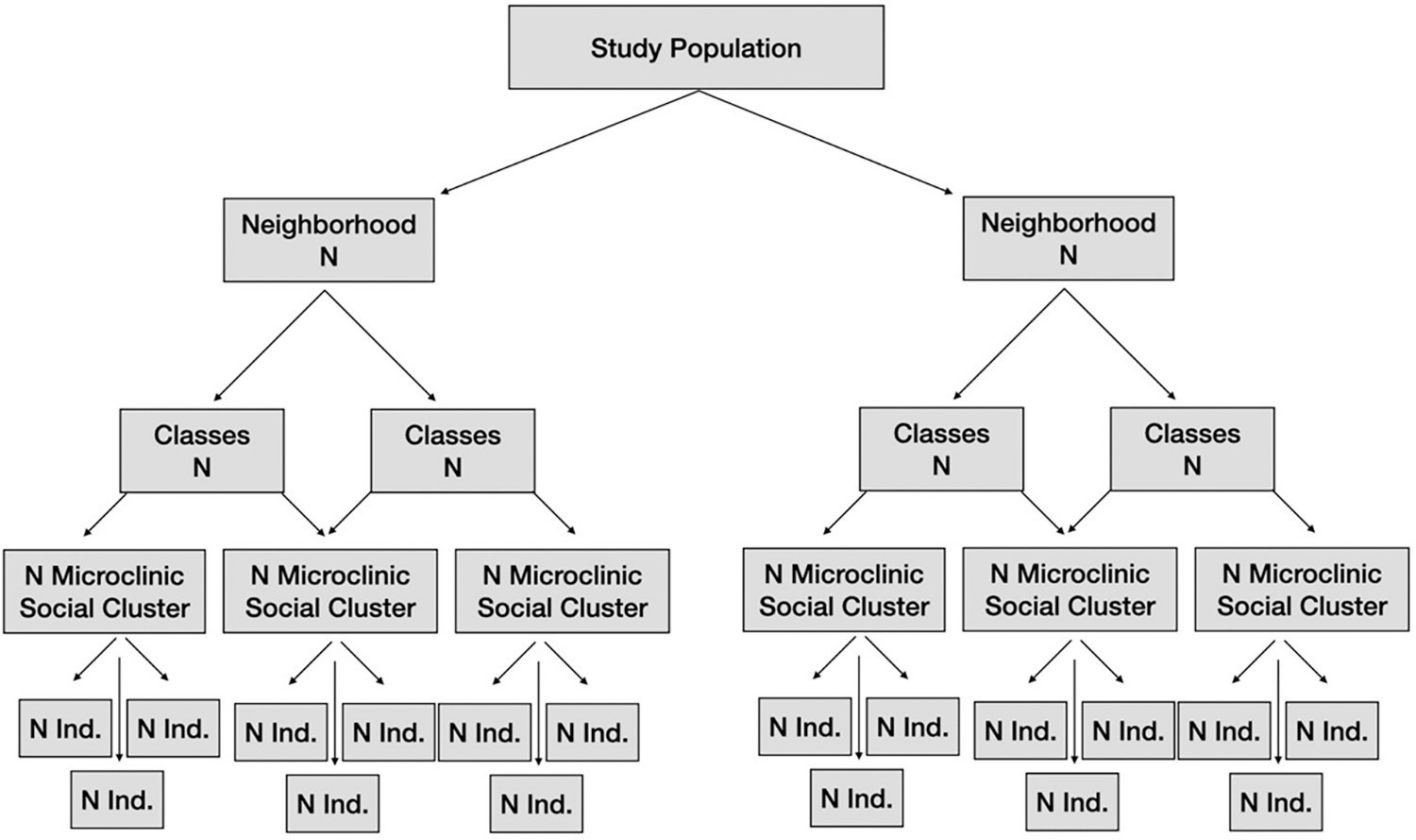
Hierarchical nesting structure.

Enrollment is described in Figs 1 and 2. Randomization of nodes was allocated with the ratio of 3:1:1, with resulting group sizes of n = 540, 186, and 188 in arms A, B, and C, respectively.

#### Data collection

Clinical data was collected by trained nurses/study personnel at MoH centers. We collected data on clinical measures (height, weight, waist circumference, BP, HbA1c, and FBG), health knowledge, behavior, and demographic data (including survey questions on diabetes/obesity knowledge, healthcare access, exercise/dietary habits, and education status). Survey and social network data were collected via paper-based surveys, administered by nurses/coordinators. Participants were required to fast before their appointment. Height was measured using a hospital and home scale with height meter; weight was measured using Health Scale SVR160. Fasting Plasma glucose was measured via finger prick using AccuCheck-Performa. Digital readings on this device converted blood glucose concentrations to plasma glucose concentrations, conforming to international reporting standards. Omron and A&D blood pressure cuffs were used to measure blood pressure; both types measured systolic blood pressure (SBP) and diastolic blood pressure (DBP) three consecutive times; mean arterial pressure was calculated using the clinical formula MAP = (SBP + (2*DBP))/3. HbA1c levels were tested at MoH Lab Centers via High Performance Liquid Chromatography (HPLC). Laboratory analysis for HbA1c was masked, meaning technicians were not aware which treatment group the samples came from. No masking took place for other outcome measurements.

#### Primary and secondary outcomes

The primary outcomes of interest were the overall changes from baseline across 2 years of follow-up of: 1) weight; 2) FBG; 3) HbA1c; and 4) BP, indexed via MAP (derived from SBP/DBP). To further understand the potential difference between variations of the Full and Basic MCP versus controls, our secondary aim evaluated the follow-up outcomes of body weight, HbA1c, FBG, and blood pressure between individual trial arms.

### Statistical analyses

We structured the trial data as a longitudinal, multi-level, panel dataset. We estimated the differences across trial arms of each endpoint using multilevel longitudinal analysis with hierarchal nesting of individuals (within MCP classrooms, within class days, within community centers, and within temporal cohort waves), and using unstructured covariance for time, and with random intercepts at each hierarchal level. The modelling was performed using the intention-to-treat principle, with relative effects in Full and Basic MCP expressed as difference-in-difference against the controls. The overall net effect across all time periods between the Full MCP and the controls and between the Basic MCP and the controls were analyzed as the joint test of all the time-by-group interaction coefficients. Because of the hierarchal social network nested structure of the study setting, and because of the major influence of Ramadan on eating and fasting in Muslim society and prior Ramadan-related body weight effects found in Jordan [28, 29], all analyses adjusted for age, sex, fasting, cohort wave, neighborhood, day of week, and external current event factors (Ramadan and political unrest), and key interactions with the external factors. We also conducted two-arm head-to-head analysis comparing individual arms, as well as stratified analysis by obesity status at baseline and by gender. We further conducted mediation analysis of the intervention by including changes in concomitant changes in diet, physical activity, glucose monitoring, and medication adherence. All statistical analyses were performed using STATA 13.1 (College Station, Texas); all P values were two-tailed.

## Results

### Participants at baseline

1025 volunteers were screened for eligibility. Of these, 78 did not consent, and 33 did not meet inclusion criteria (5 were pregnant, and 28 were neither diabetic nor pre-diabetic) (Fig 1). Thus, 914 eligible individuals were randomized to the three treatment arms. 540 were randomly assigned to Arm A and received Full MCP; 186 were randomized to Arm B and received Basic MCP; 188 were assigned to Arm C, the control arm. Via the post-randomization microclinic formation process, Arm A yielded 213 microclinics, Arm B had 141 microclinics, and Arm C had 151 microclinics.

Of the 540 participants of Arm A, 382 completed measurements at 6-months; 385 completed follow-up measurements at 2-years post baseline. Of the 186 participants in Arm B, 137 of 186 completed 6-month measurements, and 121 completed 2-year measurements. In 188 participants in Arm C, 118 completed measurements at 6 months, and 108 were present at the 2-year follow-up (Fig 1).

Study participants’ baseline characteristics across all three treatment arms were generally similar (Table 1), with mean age in Arm A being a minor exception (mean age 54.2 years versus Arm B 56.6 years, versus Arm C 56.2 years). Mean BMI was similar across the three arms (Arm A 33.6 kg/m^2^ versus Arm B 33.5 kg/m^2^ versus 33.4 kg/m^2^). Mean SBP was slightly lower in Arm A (129 mmHg) versus Arm B and the control group (both >131.7 mmHg). Mean FPG was slightly higher in Arm A (147.8 mg/dL) compared to Arm B (142.8 mg/dL) and Arm C (145.7 mg/dL).

**Table 1:** Baseline participant characteristics.

Characteristic, mean (SD) or %	Full MCP	Basic MCP	Control Group
	(n = 540)	(n = 186)	(n = 188)
Age, years	54.2	56.6	56.2
Women (%)	66.5	67.0	65.0
Weight, kg	85.9	85.0	86.0
Height, m	160.3	159.6	160.3
BMI, kg/m^2^	33.6	33.5	33.4
Obese (% BMI> = 30)	67.8	72.0	70.7
Systolic blood pressure, mm Hg	129.1	131.7	132.2
Diastolic blood pressure, mm Hg	81.3	81.0	81.3
Mean arterial pressure, mm Hg	97.2	97.9	98.3
Hypertension (%, SBP> = 140 mm Hg, or DBP> = 90 mm Hg)	17.6	16.7	18.1
HbA1c (%) (SD)	6.91	6.90	6.91
Fasting plasma glucose^a^, mg/dL	147.8	142.8	145.7

^a^Baseline fasting glucose average of first and second weeks; some participants did not provide all risk factor data at baseline.

Following CONSORT guidelines, we did not report on the p-values of the difference in baseline characteristics, based on the reasoning that any significant differences would have arisen through a random process [31].

### Three arm comparisons

In our analysis comparing the Full and Basic MCP with the controls across 2 years, we observed significant overall differences between the three groups for clinical endpoints of the change in: body weight (P = 0.0003), FBG (P = 0.0015), HbA1c (P = 0.0004), but not significant overall 3-arm differences for MAP (P = 0.45), though significant Full MCP versus controls divergence was found for mean arterial pressure (P = 0.002). Notably, we highlight some specific findings.

For weight change, in direct comparisons of the Full MCP versus controls, we found greater overall improvements with Full MCP–notably, we found that during the intervention, Full MCP yielded significantly larger weight loss of -1.51 kg (95% CI: -0.74 to -2.29) versus the control group (P<0.001) at 6 months (S1 Fig, Panel A and Table 2), while the Basic MCP did not, with a non-significant 0.54 kg decline compared to controls. However, during the 2-year follow-up, the Basic MCP’s weight trajectory caught up to the Full MCP, and both Full and Basic MCP yielded similar weight losses of 1.61 and 1.68 kg versus controls at 2 years, respectively. Both the Full MCP and Basic MCP programs yielded an overall benefit versus controls over 2 years (P value of Full MCP vs. controls<0,001; P value of Basic MCP vs. controls = 0.02). Furthermore, during the intervention period, the Arm A Full MCP intervention showed significantly superior weight loss to the Arm B Basic MCP, thereby demonstrating weight loss was enhanced by the addition of social network intervention programming.

**Table 2:** Intervention effects on body weight change (kg), over 2 years.

Session (weeks)	Full Social Network (Arm A) vs C control	95% CI lower	95% CI upper	P value for effect of A vs C across all time points (secondary pre-specified analysis)
0 (Baseline)	(ref)	-	-	
2–3	-0.31	-0.90	0.29	
5–6	-0.61	-1.25	0.03	
9–11	-1.17	-1.83	-0.52	
15	-0.85	-1.61	-0.09	
21	-1.00	-1.78	-0.22	
27**	-1.51	-2.29	-0.74	
Follow-up: 2 yr	-1.61	-2.39	-0.84	<0.001
	**Basic Social Network (Arm B) vs C control**	95% CI lower	95% CI upper	P value for effect of B vs C across all time points (secondary pre-specified analysis)
Session (weeks)				
0 (Baseline)	(reference)	-	-	
2–3	-0.32	-1.04	0.39	
5–6	-0.40	-1.16	0.37	
9–11	-0.90	-1.68	-0.11	
15	-0.49	-1.40	0.43	
21	-0.86	-1.81	0.09	
27**	-0.54	-1.46	0.38	
Follow-up: 2 yr	-1.68	-2.61	-0.74	0.02
**Primary Pre-specified Analysis:** **Global Joint P value test for inequality 3 arm over time (A, B, C)**				**0.0003**

Comparing the Full MCP versus controls for fasting glucose change, we observed that the Full MCP resulted in a significant overall reduction versus controls over the 2 year period (P<0.0001; Table 3). However, the overall effect of the Basic MCP versus controls was insignificant (P = 0.42). The Full MCP achieved a mean reduction in fasting glucose of 3.52 mg/dL (95% CI: -2.99 to 7.80) at 6 months, and a significant mean reduction of 7.04 mg/dL (95% CI: 3.52 to 9.32) (P<0.001) at 2 years. In comparison, the Basic MCP did not lead to significant improvement in fasting glucose at both 6 months (P = 0.85) and 2 years (P = 0.50).

**Table 3:** Intervention effects on fasting plasma glucose change, excluding Ramadan, over 2 years.

Session (weeks)	Full Social Network (Arm A) vs C control	95% CI lower	95% CI upper	P value for Joint test of A vs C over time (secondary pre-specified analysis)
0 (Baseline)	(ref)	-	-	
2–3	-1.50	-5.04	1.84	
5–6	-5.65	-7.62	-0.09	
9–11	-6.03	-16.56	2.04	
15	-5.87	-17.10	2.07	
21	-6.98	-15.62	1.46	
27**	-3.52	-7.80	2.99	
Follow-up: 2 yrs	-7.04	-9.32	-3.52	<0.0001
	**Basic Social Network (Arm B) vs C control**	95% CI lower	95% CI upper	P value for Joint test of B vs C over time (secondary pre-specified analysis)
Session (weeks)				
0 (Baseline)	(reference)	-	-	
2–3	2.77	-8.45	13.99	
5–6	-2.59	-6.51	1.34	
9–11	-2.98	-7.04	1.09	
15	-3.84	-19.99	12.31	
21	-1.13	-17.14	14.89	
27**	0.84	-7.89	9.57	
Follow-up: 2 yr	-4.45	-17.40	8.50	0.42
**Primary Pre-specified Analysis:** **Global Joint P value test for inequality 3 arm over time (A, B, C)**				**0.0015**

For percentage change in HbA1c, both Full MCP and Basic MCP led to significant overall improvement over the 2 years period versus controls (P<0.0001 for both Full MCP and Basic MCP) (S1 Fig, Panel C and Table 4). The Full MCP led to a significant reduction in HbA1c at both 6 months (P<0.001) and 1 year follow-up (P<0.001), with a mean reduction of 0.31 (95% CI: 0.14 to 0.47) at 6 months and a mean reduction of 0.19 (95% CI: 0.14 to 0.23) at 1 year. On the other hand, the effect of the Basic MCP versus controls was not found to be significant at both 6 months (P = 0.449) and 1 year follow-up (P = 0.886), with a mean reduction of 0.09 (95% CI: -0.14 to 0.32) at 6 months and 0.02 (95% CI: -0.22 to 0.25) at 1 year, respectively. However, Full MCP and Basic MCP did not lead to improvement in HbA1c relative to controls at 2 years follow-up (P = 0.964 for Full MCP and P = 0.168 for Basic MCP).

**Table 4:** Intervention effects on HbA1c change, over 2 years.

Session (weeks)	Full Social Network (Arm A) vs C control	95% CI lower	95% CI upper	P value for Joint test of A vs C over time (secondary pre-specified analysis)
0 (Baseline)	(ref)	-	-	
15	-0.51	-0.82	-0.20	
27**	-0.31	-0.47	-0.14	
Follow-up: 1 yr	-0.19	-0.23	-0.14	
Follow-up: 2 yr	0.01	-0.23	0.24	<0.0001
	**Basic Social Network (Arm B) vs C control**	95% CI lower	95% CI upper	P value for Joint test of B vs C over time (secondary pre-specified analysis)
Session (weeks)				
0 (Baseline)	(reference)	-	-	
15	-0.36	-0.51	-0.20	
27**	-0.09	-0.32	0.14	
Follow-up: 1 yr	-0.02	-0.25	0.22	
Follow-up: 2 yr	0.34	-0.14	0.82	<0.0001
**Primary Pre-specified Analysis:** **Global Joint P value test for inequality 3 arm over time (A, B, C)**				**0.0004**

For MAP, the effects of the Full and Basic MCP relative to the controls are shown in S1 Fig, Panel D and Table 5. We observed that both Full and Basic MCP yielded a greater drop in BP especially at 6 months, with a mean reduction of 3.78 mmHg (95% CI: -1.53 to 9.10) versus controls for Full MCP, and with a mean reduction of 1.91 mmHg (95% CI: -4.54 to 8.37) versus controls. However, the reductions in mean arterial BP for both Full and Basic MCP relative to the controls were minimal at 2 years follow-up relative to the controls, with a mean reduction of 0.10 mmHg (95% CI: -2.21 to 2.24) for Full MCP, and with a mean reduction of 0.63 mmHg (95% CI: -3.24 to 4.51) for Basic MCP.

**Table 5:** Intervention effects on mean arterial blood pressure change, over 2 years.

Session (weeks)	Full Social Network (Arm A) vs C control	95% CI lower	95% CI upper	P value for Joint test of A vs C over time (secondary pre-specified analysis)
0 (Baseline)	(ref)	-	-	
2–3	-2.61	-5.55	0.32	
5–6	-0.61	-5.45	4.23	
9–11	-1.84	-4.15	0.48	
15	-0.51	-4.44	3.43	
21	-0.90	-2.92	1.12	
27**	-3.78	-9.10	1.53	
Follow-up: 2 yr	-0.10	-2.42	2.21	0.002
	**Basic Social Network (Arm B) vs C control**	95% CI lower	95% CI upper	P value for Joint test of B vs C over time (secondary pre-specified analysis)
Session (weeks)				
0 (Baseline)	(reference)	-	-	
2–3	-3.14	-5.61	-0.67	
5–6	-1.65	-3.99	0.68	
9–11	-2.02	-4.13	0.09	
15	-0.05	-4.53	4.42	
21	-1.69	-4.01	0.64	
27**	-1.91	-8.37	4.54	
Follow-up: 2 yr	-0.63	-4.51	3.24	0.36
**Primary Pre-specified Analysis:** **Global Joint P value test for inequality 3 arm over time (A, B, C)**				**0.45**

** Week 27 was the end of 6-month active intervention program.

### Two arm comparisons

During the intervention period, Arm A Full MCP intervention showed significantly superior weight loss compared to the Arm B Basic MCP program (-0.97 kg, 95% CI: -0.40 to -1.54; P<0.001), thereby demonstrating weight loss was enhanced by the specific addition of social network intervention programming. However, even though both Full MCP and Basic MCP were superior versus Controls, fasting glucose, HbA1C, and MAP did not show significant difference between Full MCP and Basic MCP groups in direct comparison—which may be because there was some cross-pollination between Arms A and B. Furthermore, no weight differences in A and B were seen for weight at 2-year follow-up, after the intervention is over—possibly due to people in various arms living in the same community intermixing, thereby cross diluting many effects between arms during follow-up.

### Gender analysis

We also conducted gender-stratified results for weight, presented in Table 6. While some minor differences were seen, with men losing more weight in the first few months of the MCP intervention than women, weight loss between genders were similar long term at 6 months and 2 years. Overall results showed the Full MCP intervention was effective for weight loss in both genders.

**Table 6:** Gender-stratified results: Social network intervention effects on body weight change (kg), over 2 years*, in women and men.

Gender	Women			Men			
Session (weeks)	Full Social Network (Arm A) vs C control	95% CI lower	95% CI upper	Full Social Network (Arm A) vs C control	95% CI lower	95% CI upper	P value for Gender effect modification
0 (Baseline)	(ref)	-	-				
2–3	-0.21	-0.84	0.42	-0.18	-1.21	0.85	
5–6	-0.18	-0.87	0.51	-1.07	-2.18	0.03	
9–11	-0.83	-1.52	-0.14	-1.33	-2.47	-0.19	
15	-0.54	-1.35	0.27	-1.12	-2.44	0.19	
21	-0.81	-1.65	0.03	-1.00	-2.33	0.33	
27**	-1.27	-2.11	-0.43	-1.59	-2.90	-0.27	
Follow-up: 2 yr	-2.52	-3.83	-1.20	-2.11	-4.04	-0.19	
							0.0003

### Mediator analysis by concomitant changes in diet, physical activity, monitoring, and medication

When taking into account the mediation effect of diet, direct effect of full MCP relative to control was reduced by 0.3%, with an estimated effect of -3.39 kg (-5.60 to -1.19). When taking into account the mediation effect of physical activity, direct effect of full MCP was reduced by 15%, with an estimated effect of -2.89 kg (-5.01 to -0.77). When taking into account the mediation effect of medication, effect of full MCP was reduced by 5.3%, with an estimated effect of -3.22 kg (-5.28 to -1.17). When accounting for the mediation effect of monitoring behaviour, the direct effect of full MCP relative to control was reduced by 14.7%, with an estimated effect of -2.90 kg (-4.74 to -1.06). When accounting for the mediation effect of monitoring resources, the direct effect of full MCP relative to control was reduced by 15.9%, with an estimated effect of -2.86 kg (-4.45 to -1.29). When accounting for the mediation effects of diet, physical activity, and monitoring behavior, the direct effect of full MCP was reduced by 9.7%, with an estimated effect of -3.07 kg (-5.50 to -0.65). When accounting for mediation effects of diet, physical activity, monitoring behavior, monitoring resources, and medication, the direct effect of full MCP was reduced by 12.1%, with an estimated effect of -2.99 kg (-5.31 to -0.67). We observed comparable reductions in the effects of basic MCP relative to controls after adjusting for mediation factors (S1 Table).

### Effect modification by baseline obesity status

The magnitude of the intervention’s effect on weight loss was substantially greater in those not obese at baseline, -2.24 kg (-4.23 to -0.26) for Full MCP and -1.66 kg (-3.40 to 0.08) for Basic MCP, than those with baseline obesity, -1.82 kg (-2.33 to -1.30) for Full MCP and -0.36 kg (-2.69 to 1.98) for Basic MCP (Table 7).

**Table 7:** Intervention effects on body weight change (kg), over 2 years.

(Change from baseline)	Group B vs. Control	Group A vs. Control	P for effect modification
	Basic MCP Intervention versus Control	Full MCP Intervention versus Control	
	(Weight change, 95% CI)	(Weight change, 95% CI)	
Weight Loss, kg			P<0.001
Non-Obese (BMI<30)	-1.66 (-3.40 to 0.08)	-2.24 (-4.23 to -0.26)	
Obese (BMI> = 30)	-0.36 (-2.69 to 1.98)	-1.82 (-2.33 to -1.30)	
Weight Loss, % change			P<0.001
Non-Obese (BMI<30)	-1.88 (-4.97 to 1.21)	-2.73 (-5.83 to 0.36)	
Obese (BMI> = 30)	-0.60 (-2.39 to 1.20)	-2.24 (-2.85 to -1.62)	

### Sensitivity analyses

The study’s follow-up retention rates at the end of the intervention were 70.7% in Full MCP group, 73.7% in the Basic MCP group, and 62.8% in the control group. At the 2-year follow up, participant follow-up return rates in our low-income communities with refugee populations were still 71.3% for Full MCP, 65.1% for Basic MCP, and 57.4% for the control group. We further describe a sensitivity analysis for missingness at follow-up in S2 Appendix, which showed the intervention results appeared significant even accounting for dropout.

## Discussion

Overall, we found that both the Full and Basic MCP yielded sustained weight loss improvements over 2 years versus controls. For HbA1c, both Full and Basic MCP were superior to the controls with long-term attenuation. To a lesser extent, both Full and Basic MCP were found to be superior to control in terms of MAP. The mediator analysis suggests physical activity, monitoring, medication, and dietary behavior change hold different levels of importance and should be studied.

The direct comparison of the Full MCP versus Basic MCP demonstrates conclusively that weight loss was enhanced by the addition of social network programming. However, the discrepancy between weight and other outcomes could be explained by the greater overall weight loss seen in Full MCP versus Basic MCP. Also, cross-pollination in closely knit neighborhoods likely enabled Basic MCP to catch up with Full MCP, as study subjects in various arms start to mix and spread knowledge and behaviors in the same neighborhood.

Results presented are clinically significant with each kilogram of weight loss reducing progression to type-2 diabetes by 16%, and, weight improvements at any level yielding health benefits [32, 33]. Our results are also comparable to weight loss induced by a pharmacological (metformin) intervention undertaken during a similar time-period, albeit in a well-resourced setting [34]. Therefore, these results support the theory that activated social networks can be harnessed to cost-effectively strengthen positive health behaviors and improve outcomes.

### Social networks for preventive interventions

Social network interactions may play a meaningful role in lifestyle modification for prevention and management of chronic diseases [35–38]. Evidence indicates that social network mechanisms are a major driver of patterns of lifestyle and risk factors such as smoking and alcohol use that contribute to obesity and metabolic risks [14, 39, 40] often to multiple degrees of social network separation.

Since behaviors may naturally aggregate and spread within social networks, interventions that leverage social networks may hold potential to alter behavior-shaping norms via mechanisms of social support, social influence, and possibly even large-scale social change [35, 36, 39, 41, 42].

While some lifestyle programs have involved social support and group-based interventions [43, 44], few have tested the causal effects of structured or informal social support on obesity and diabetes outcomes, and none have done so in a Middle Eastern setting [14, 36, 39]. Moreover, existing social network-based studies are often limited in scope. For instance, Vissenberg et al. (2017) recognizes the intrinsic importance of social networks in self-management programs to create sustainable health behavior change among enrolled participants; however much of the literature on the topic is passive, observational, and hence limited by the conclusions they can draw [14, 17, 24, 39, 40, 45, 46]. The evidence presented here addresses this gap. It highlights the potential of leveraging social networks in propagating healthy lifestyles and in affecting large-scale, sustained, low-cost behavior change, which is key in successfully combatting chronic diseases in emerging economies.

### Limitations

This study had several limitations, including a high dropout rate, but one that is comparable in proportion to another leading trial. This was impacted by several factors stemming from the fact that the intervention was implemented in low-income and multi-generation refugee communities formed during various waves of regional conflict. For example, local study staff reported that the use of widespread prepaid cell phones with changing numbers made it difficult to track down patients. Furthermore, tracing participants via mail proved difficult since mailing addresses were only established midway through the study.

Individuals enrolled in the program predominately received medical care at either a Jordanian Ministry of Health-run medical clinic and/or from the United Nations Relief Works Agency network of clinics for refugees. Due to broader socio-economic, environmental, and political conditions in the country during the study, data collection was not without limitations, hindering full follow-up of every participant. In particular, the data collection for the 12-month follow-up was disrupted by extreme weather events and geo-political instability. Thus, they were inconsistently collected and consequently discarded.

Another study limitation is that most participants were female, suggesting that future iterations of the intervention need to take into consideration differences in the unique daily schedules of men and women, as well as cultural norms. In some cultures, women may have more home-based responsibilities, making it possible for them to attend neighborhood classes, even with small children. Men may engage in professions such as driving taxis and construction, which are less flexible. For some, there may also be some sensitivities around discussing body weight around members of the opposite sex. Fortunately, we had mixed-gender classes for all participants in all trial arms—they may explain why we did not observe significant gender differences of effect between arms.

Although trial retention rates were similar between Full MCP and Basic MCP groups at 6 months (71–73%), the controls had slightly lower follow-up rates at both ends of intervention (63%) and 2-year (58%) follow-ups. However, our trial’s retention rates in all arms were substantially higher than the CDC’s flagship Diabetes Prevention Program (DPP) retention rates [47] at 18 weeks follow-up (63%), and at 44 weeks follow-up (32%)—which means that CDC’s renowned DPP’s 10-month retention rate is in fact roughly half the retention rates of our study’s 2-year follow-up. While lower follow-up rates in controls is often expected in weight loss trials in general, the observed weight loss effects of Full MCP versus controls remained significant in missingness-adjusted models (briefly described in S2 Appendix). Furthermore, because it is possible that participants in the control group may perceive fewer benefits to continue their long-term participation, and often those who don’t return may have seen lesser weight loss—this could also mean that the true weight loss difference between MCP participants versus controls (had all controls returned) could have been even wider and larger in weight disparity at the end of the trial. Therefore, our results may possibly be conservative.

Another study characteristic–“cross pollination”–may be initially viewed as a limitation; but it in fact, serves only to bolster our findings. Virtually any social network-based RCT, especially one operated in close-knit Middle Eastern communities, will have some level of social "cross pollination” through interactions in online social groups, social clubs, shops, family functions, and places of worship. These cross-pollination interactions can make controls look more like intervention participants and vice versa. However, this is a likely reality of true community social networks, and actually suggests that arms A and B may had even greater positive effects than that observed here; thus, our attenuated results may again be a conservative underestimate of more profound impacts of social networks.

Finally, while many results were presented and subject to chance finding due to multiple comparisons, all 4 of our primary clinical endpoints were pre-specified. Further, although there were many time periods, a single overall global test for joint intervention effect differences across all time periods was parsimonious and presented for each of the 4 clinical measures—the significant ones of which were also still significant even if one performed Bonferroni correction for multiple comparisons.

## Conclusion and policy implications

This study tested the MCP on a low-income and refugee population in the Middle East. This contributes to knowledge about the MCP effect in different geographical areas, extending generalizability beyond the low-income communities it was previously tested in (e.g., Kentucky, other neighborhoods in Jordan, Lebanon, West Bank, and Gaza) [21, 27]. Initial insights contributed to successful efforts to scale the model at regional, national, or transnational levels by Queen Rania’s Royal Health Awareness Society [48], the Jordanian Ministry of Health [49], the Mexican Ministry of Health [50, 51], Kenya Medical Research Institute [26], and the United Nations Relief Works Agency [27, 52]. Further work is needed to determine how MCP could be integrated into other health systems, regionally and globally. Additionally, further studies are needed to enhance and accelerate cardio-metabolic improvements in the long-term, perhaps through extended virtual or in-person engagement.

## Supporting information

S1 Table MSNP social structure and curriculum comparisons. (DOCX)

S2 Table Intervention schedule, highlighting differences between Arm A and Arm B curriculum. (DOCX)

S3 Table Mediation analysis of the intervention effects on body weight change, by concomitant changes in diet, physical activity, medication, diabetes monitoring behavior, and monitoring resource. (DOCX)

S1 Fig Change in metabolic risk factors, over time, by intervention group. Panel A in S1 Fig. Change in Weight (kg). Panel B in S1 Fig. Change in Fasting Glucose (mg/dL), Excluding Ramadan. Panel C in S1 Fig. Change in HbA1c. Panel D in S1 Fig. Change in Mean Arterial Pressure (mmHg).
(DOCX)

S1 Appendix Power calculations. (DOCX)

S2 Appendix Sensitivity analysis for missingness at follow-up. (DOCX)
