# Supplementary material for: Social network enhanced behavioral interventions for diabetes and obesity: A 3 arm randomized trial with 2 years follow-up in Jordan
Source: PLOS Glob Public Health. 2024 Mar 20;4(3):e0001514. doi: 10.1371/journal.pgph.0001514 (PMC10954161; doi:10.1371/journal.pgph.0001514)
Supplement: S1 Table — (DOCX) [file pgph.0001514.s001.docx]

**S1 Table. MSNP social structure and curriculum comparisons**

***MSNP Curriculum Design comparing all 3 trial arms***

| Intervention Features | Arm A (Full MCP) | Arm B (Basic MCP) | Arm C |
| --- | --- | --- | --- |
| Parallel Measurements | X | X | X |
| Diabetes Education | X | X |  |
| Friends/Family secondaries who participated in support | X |  |  |
| Goal Setting | X | X |  |
| Social Network Programming | X |  |  |
