# Supplementary material for: Social network enhanced behavioral interventions for diabetes and obesity: A 3 arm randomized trial with 2 years follow-up in Jordan
Source: PLOS Glob Public Health. 2024 Mar 20;4(3):e0001514. doi: 10.1371/journal.pgph.0001514 (PMC10954161; doi:10.1371/journal.pgph.0001514)
Supplement: S2 Table — (DOCX) [file pgph.0001514.s002.docx]

**S2 Table. Intervention schedule, highlighting differences between Arm A and Arm B curriculum**

| **Session*** | **Group A curriculum**  **(Directed social interactions)** | **Group B curriculum**  **(Organic social interactions)** |
| --- | --- | --- |
| 1-Baseline, Orientation to program philosophy | -Ice breaker: participants go around the room and introduce themselves by way of interesting anecdotes.  -Team-building: participants perform team building activities.  -Program philosophy: participants are introduced to the program’s social aims, i.e. to recognize how they affect the behavior of others, how this can be both good or bad, and how they can make it a good thing by helping others eat healthier.  -Participants are introduced to 4 M’s as the key behavior change targets: (Medication, Monitoring, Movement, Meals) | -No ice breaker  -Participants receive a brief introduction to the program.  -Participants are introduced to 4 M’s as the key behavior change targets: (Medication, Monitoring, Movement, Meals) |
| 2-Diabetes Overview | -Ice breaker: participants go around the room and get to know one another.  -Group and individual goal setting: participants learn how to establish behavioral targets for themselves and with their larger circle of family and friends. These goals target the four M’s  -Group Activities: these activities involve in-class groupwork focused on the four Ms  -Homework: meet with group and go for walk | -No ice breaker  -Individual goal setting: participants learn how to establish behavioral targets for themselves. These goals target the four M’s.  -Homework: go for a walk. |
| 3-Monitoring Hypo/Hyperglycemia | -Ice breaker: participants go around the room and get to know one another  -Set up: class sits in a circle  -Group and individual goal setting: participants learn how to establish behavioral targets for themselves and with their larger circle of family and friends. These goals target the four M’s  -Homework: 2 individual and 1 MC goal | -No ice breaker.  -Class sits facing forward.  -Homework: 2 monitoring goals to complete on their own. |
| 4-Nutrition for Diabetic Individuals | -Ice breaker: participants go around the room and get to know one another  -Portion size group activity: participants work together to learn about what a healthy plate looks like for a diabetic individual  -Class breaks up into MCs and talk about their goals for the week | -No ice breaker  -Homework: 2 monitoring goals on their own  -Class is given a handout and a lecture on healthy portion sizes |
| 5-Importance of Walking | -Ice breaker: participants go around the room and get to know one another  -Physical Activity Lecture: the class is started by engaging with participants concerning the benefits are of walking  -Market visit: participants go on a “field trip” to the local food market  -Class discusses progress towards goals with MCs  -Homework: individual and MC goal  -Walking competition established between small groups | -No ice breaker  -Lecture: facilitator goes straight to explaining the benefits of walking  -No market visit  -Homework: 2 individual goals |
| 6-Diabetes medication | -Ice breaker: participants go around the room and get to know one another  -Class sits in circle  -Lecture on diabetes medication starts with asking the class a question  -Diabetes Medical Activity: class breaks up into MCs and discuss their experiences with oral medication and insulin shots. Then the instructor demonstrates how to take insulin  -Class discusses progress towards goals with MCs | -No ice breaker  -Class sits facing forward  -Lecture on diabetes medication starts with explanation on types of medication for diabetes  -Diabetes Medical Activity: instructor demonstrates how to take insulin |
| 7-Nutritionist vegetable variety cooking class | -Ice breaker: participants go around the room and get to know one another  -Cooking demonstration: participants are given two options: 1 is to do an interactive cooking demo using some participants as assistant chefs, followed by tasting and discussion; 2 is to watch a video of a cooking demo and discuss  -Participants engage in role-playing group activity on strategies to refuse food  -Q&A with the nutritionist  -Discuss progress towards goals with MCs  -Homework: individual and MC goal | -No ice breaker  -Cooking demo -- participants are given two options: 1 lecture the different ways to make traditional dishes healthy; 2 is to just watch the video  -Lecture: refusing food  -Lecture with nutritionist. Nutritionist just covers common myths and questions that were previously compiled.  -Homework: Set 1 individual goal |
| 8-Diabetes Complications | -Ice breaker: participants go around the room and get to know one another  -During lecture, have a group discussion about the ways in which diabetes complications have affected individuals and their families  -Activity on footcare performed on participants | -Lecture on diabetes complications  -Instead of participants doing the footcare activity, the instructors do it in front of the class using a volunteer |
| 9-Physical Activity | -Ice breaker: participants go around the room and get to know one another  -Team-building activity: participants work on groups on activity inspired by the lectures  -Class actively participates in a calisthenics routine and receives a handout with exercise instructions for use at home  -Discuss progress towards goals with MCs  -Homework: individual and MC goal | -No ice breaker  -The class will just sit and watch the instructor demonstrating calisthenics routine and give out handout so they can do it at home  -Homework: individual goal |
| 10-Midpoint, Diabetes Myths, and Abu Salem video | -Ice breaker: participants go around the room and get to know one another  -Class is separated into groups; each write a list of diabetes myths, and these are discussed collectively  -Abu Salem health awareness cartoon video is viewed and discussed  - Discuss progress towards goals with MCs  -Homework: individual and MC goal | -Instructor goes over diabetes myths in a lecture format  -Participants watch the Abu Salem health cartoon video  -Homework: set individual goal |
| 11-Eye Exam | -Ice breaker: participants go around the room and get to know one another  -Team-building activity: sharing wisdom about diabetes  -Eye exam with short Q&A and discussion to follow.  - Discuss progress towards goals with MCs  -Homework: individual and MC goal | -Eye exam where the provider shares some common Q&A but no discussion is moderated  -Homework: individual goal |
| 12-Nutrition review | -Ice breaker: participants go around the room and get to know one another  -Sit in a circle during lecture about nutrition  -Team-building activity about dealing with pressure  --Discuss progress towards goals with MCs  -Homework: individual and MC goal | -Sit facing instructor during lecture  -Homework: individual goal setting |
| 13-Field trip to the gym | Field trip to the gym  -Discuss progress towards goals with MCs  -Homework: individual and MC goal | Field trip to the gym  -Homework: individual goal setting |
| 14-Final class | -Potluck and MC presentations of their collective goals  -Participant certificates | -Participant certificates |

*Neighborhood reunions for program participants were held approximately 2-3 months prior to the 2 year follow-ups.
