# Supplementary material for: Social network enhanced behavioral interventions for diabetes and obesity: A 3 arm randomized trial with 2 years follow-up in Jordan
Source: PLOS Glob Public Health. 2024 Mar 20;4(3):e0001514. doi: 10.1371/journal.pgph.0001514 (PMC10954161; doi:10.1371/journal.pgph.0001514)
Supplement: S3 Table — (DOCX) [file pgph.0001514.s003.docx]

**S3 Table. Mediation analysis of the intervention effects on body weight change, by concomitant changes in diet, physical activity, medication, diabetes monitoring behavior, and monitoring resource**

| OUTCOME  (Change from baseline) | Arm B vs. Control  Basic MCP Intervention vs. Control  (effect, 95% CI) | Attenuation from BASE model of Arm B vs. Control | Arm A vs. Control  Full MCP Intervention vs. Control  (effect, 95% CI) | Attenuation from BASE model of Arm A vs. Control |
| --- | --- | --- | --- | --- |
|  |  |  |  |  |
| Weight, kg |  |  |  |  |
|  |  |  |  |  |
| Base model* | -1.51 (-1.73 to -1.30) | [reference] | -3.40 (-6.02 to -0.77) | [reference] |
| Base + Diet | -1.49 (-1.87 to -1.11) | -1.3% | -3.39 (-5.60 to -1.19) | -0.3% |
| Base + Physical Activity | -1.27 (-1.90 to -0.63) | -15.9% | -2.89 (-5.01 to -0.77) | -15.0% |
| Base + Medication | -1.70 (-2.02 to -1.39) | n/a | -3.22 (-5.28 to -1.17) | -5.3% |
| Base + Monitoring Behavior | -1.23 (-1.48 to -0.98) | -18.5% | -2.90 (-4.74 to -1.06) | -14.7% |
| Base + Monitoring Resources | -1.32 (-1.52 to -1.13) | -12.6% | -2.86 (-4.45 to -1.29) | -15.9% |
|  |  |  |  |  |

*among those not missing diet
