# Supplementary material for: Social network enhanced behavioral interventions for diabetes and obesity: A 3 arm randomized trial with 2 years follow-up in Jordan
Source: PLOS Glob Public Health. 2024 Mar 20;4(3):e0001514. doi: 10.1371/journal.pgph.0001514 (PMC10954161; doi:10.1371/journal.pgph.0001514)
Supplement: S1 Fig — Panel A in S1 Fig. Change in Weight (kg). Panel B in S1 Fig. Change in Fasting Glucose (mg/dL), Excluding Ramadan. Panel C in S1 Fig. Change in HbA1c. Panel D in S1 Fig. Change in Mean Arterial Pressure (mmHg). (DOCX) [file pgph.0001514.s004.docx]

**S1 Fig.** **Change in metabolic risk factors, over time, by intervention group**

**Panel A: Change in Weight (kg)**

**Panel B: Change in Fasting Glucose (mg/dL), Excluding Ramadan**

**Panel C. Change in HbA1c**

**Panel D. Change in Mean Arterial Pressure (mmHg)**
