# Supplementary material for: Social network enhanced behavioral interventions for diabetes and obesity: A 3 arm randomized trial with 2 years follow-up in Jordan
Source: PLOS Glob Public Health. 2024 Mar 20;4(3):e0001514. doi: 10.1371/journal.pgph.0001514 (PMC10954161; doi:10.1371/journal.pgph.0001514)

**S1 Appendix. Power calculations**

Sample power calculations for HbA1c and weight change were performed based on sample size n=500 and estimated effects from pilot Microclinic studies. Conservative power simulations show that our study has over 90% power for less than 2.06% change in weight, and over 80% power for a 0.5% reduction in HbA1c. Additional sample power calculations were performed for HbA1c and weight change by varying the effect magnitudes (Fig 2). ***Sample Power Calculation for HbA1c and Weight Change:***


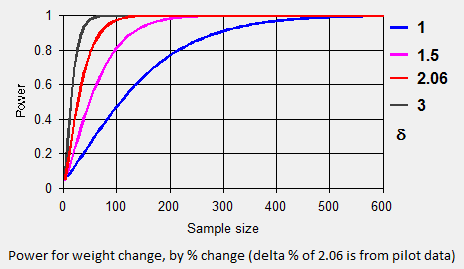

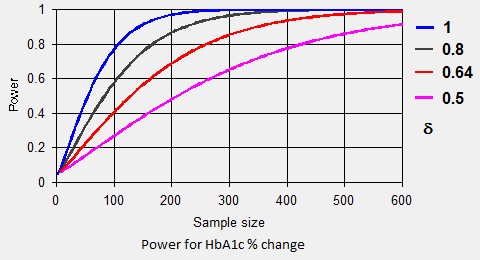

Supplement: S1 Appendix — (DOCX) [file pgph.0001514.s005.docx]
