# Supplementary material for: Social network enhanced behavioral interventions for diabetes and obesity: A 3 arm randomized trial with 2 years follow-up in Jordan
Source: PLOS Glob Public Health. 2024 Mar 20;4(3):e0001514. doi: 10.1371/journal.pgph.0001514 (PMC10954161; doi:10.1371/journal.pgph.0001514)
Supplement: S2 Appendix — (DOCX) [file pgph.0001514.s006.docx]

**S2 Appendix. Sensitivity analysis for missingness at follow-up**

Because results based on a complete case ITT analysis may be non-random and correlated with covariates and outcome, and thus possibly subject to selection bias from missingness, we also performed inverse probability-of-missingness weights (IPMW) analyses to adjust for the loss to follow up for weight to help remove missingness selection bias. The IPMW method assigns a weight to each non-missing individual and allows them to ‘represent’ those who have been lost to follow-up after adjusting for observed patient characteristics.[1-5]

We generated IPMW weights from a baseline prediction model of future non-missingness, adjusting for major demographic and trial characteristics, as well as for baseline body weight. Stabilized IPMW weights were used in the optimal model. Adjusting for missed examinations at 6 months end of intervention via IPMW, the results for weight change in Arm A versus Arm C were similar to the results obtained via complete case-ITT: and were statistically significant at 6 months. The IPWM adjusted results for weight change in Arm B versus Arm C were similar to the original results in the missingness prediction models not accounting for baseline weight, but not significant in the full missingness prediction models accounting for baseline weight. Furthermore, in all our IPMW-adjusted analyses, the overall multiple-arm test of A-B-C weight divergence at 6 months was again highly significant in all models (p<0.001). Thus, our intervention results, especially for Arm A vs Arm C, remained robust even after accounting for follow-up missingness.

**S2 Appendix Table A. Missingness adjustment via inverse probability weights: Body weight at 6 months**

| Body Weight Change at 6 months: | Arms A versus C  Weight difference (95% CI) | Arms B vs. C  Weight difference (95% CI) | P value for joint test of Arms A, B, C |
| --- | --- | --- | --- |
| Original model results (via Table 2) | -1.51 (-2.29 to -0.74) | -0.54 (-1.46 to 0.38) | P<0.001 |
| Model 1: Missingness adjusted with raw IPMW weights, without baseline body weight prediction | -1.50 (-2.00 to -1.00) | -0.54 (-0.92 to -0.16) | P<0.001 |
| Model 2: Missingness adjusted with Stabilized IPMW weight, IPMW prediction model not adjusted for baseline body weight | -1.51 (-2.02 to -1.00) | -0.54 (-0.92 to -0.17) | P<0.001 |
| Model 3: Missingness adjusted with Stabilized IPMW weights, IPMW prediction model adjusted for baseline body weight | -1.42 (-1.74 to -1.10) | 0.07 (-1.28 to 1.43) | P<0.001 |

**S2 Appendix References**

1. Hernán, M.A., B. Brumback, and J.M. Robins, *Marginal structural models to estimate the causal effect of zidovudine on the survival of HIV-positive men.* Epidemiology, 2000. **11**(5): p. 561-70.

2. Hernán, M.A. and S. Hernández-Díaz, *Beyond the intention-to-treat in comparative effectiveness research.* Clin Trials, 2012. **9**(1): p. 48-55.

3. Robins, J., *Marginal structural models versus structural nested models as tools for causal inference*, in *Epidemiology: The Environment and Clinical Trials*, B.D. Halloran M, Editor. 1999, Springer: New York. p. 95-134.

4. Robins, J.M., M.A. Hernán, and B. Brumback, *Marginal structural models and causal inference in epidemiology.* Epidemiology, 2000. **11**(5): p. 550-60.

5. Robins, J.M., A. Rotnitzky, and L.P. Zhao, *Analysis of Semiparametric Regression Models for Repeated Outcomes in the Presence of Missing Data.* Journal of the American Statistical Association, 1995. **90**(429): p. 106-121.
